# Sunscreen Ingredient Octocrylene’s Potency to Disrupt Vitamin D Synthesis

**DOI:** 10.3390/ijms231710154

**Published:** 2022-09-05

**Authors:** Sayed Aliul Hasan Abdi, Amena Ali, Shabihul Fatma Sayed, Sumathi Nagarajan, Abutahir, Prawez Alam, Abuzer Ali

**Affiliations:** 1 Department of Pharmacy, Al Baha University, Al Baha 1988, Saudi Arabia; 2 Department of Pharmaceutical Chemistry, College of Pharmacy, Taif University, P.O. Box 11099, Taif 21944, Saudi Arabia; 3 College of Nursing, University College Farasan Province, Jazan University, Jazan 54943, Saudi Arabia; 4 Hakikullah Chaudhary College of Pharmacy, Gharighat, Gonda 271312, India; 5 Department of Pharmacognosy, College of Pharmacy, Prince Sattam bin Abdulaziz University, P.O. Box 173, Al-Kharj 11942, Saudi Arabia; 6 Department of Pharmacognosy, College of Pharmacy, Taif University, P.O. Box 11099, Taif 21944, Saudi Arabia

**Keywords:** octocrylene, vitamin D disruption, sunscreen’s ingredient, in silico study

## Abstract

Octocrylene is a widely used ingredient in sunscreen products, and it has been observed that the use of sunscreen has been increasing over the last few decades. In this paper, we investigated the way in which sunscreen’s ingredient octocrylene may disrupt normal vitamin D synthesis pathway, resulting in an imbalance in vitamin D levels in the body. The key techniques used for this insilico investigation were molecular docking, molecular dynamic (MD) simulation, and MMPBSA-based assessment. Vitamin D abnormalities have become very common in human health. Unknown exposure to chemicals may be one of the important risk factors. In molecular docking analysis, octocrylene exhibited a binding energy of −11.52 kcal/mol with vitamin D binding protein (1KXP) and −11.71 for the calcitriol native ligand. Octocrylene had a binding potency of −11.152 kcal/mol with the vitamin D receptor (1DB1), and calcitriol had a binding potency of −8.73 kcal/mol. In addition, octocrylene has shown binding energy of −8.96 kcal/mol with CYP2R1, and the calcitriol binding energy was −10.36 kcal/mol. Regarding stability, the root-mean-square deviation (RMSD), the root-mean-square fluctuation (RMSF), the radius of gyration, hydrogen bonding, and the solvent-accessible surface area (SASA) exhibited that octocrylene has a stable binding pattern similar to calcitriol. These findings revealed that incessant exposure to octocrylene may disrupt normal vitamin D synthesis.

## 1. Introduction

**Figure 1:**
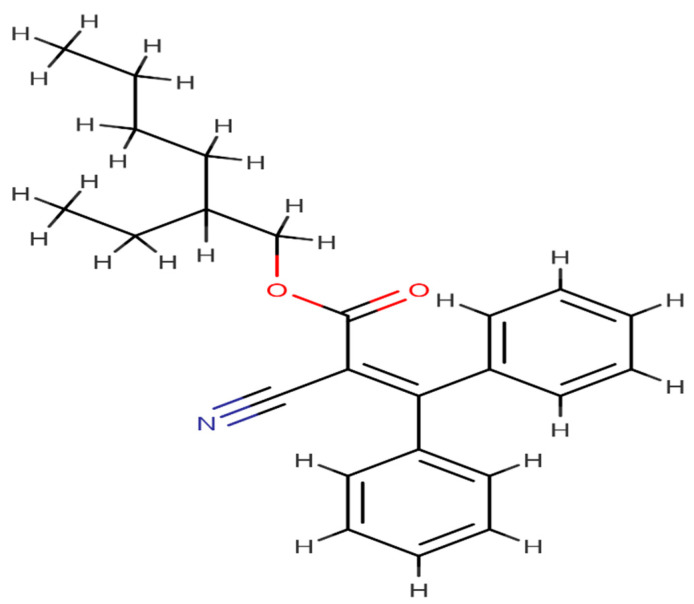
Chemical structure of octocrylene.

Octocrylene is an organic molecule that is exploited as a sunscreen and cosmetic component (Figure 1). It is an ultraviolet (UV) radiation absorber that is oil soluble and water resistant. In addition, octocrylene is very stable, and it may preserve and augment other UV absorbers while enhancing their skin coating uniformity [1]. Octocrylene may be easily absorbed into the skin and enhance reactive oxygen species [2]. However, as per the study of Matsuoka et al. (1990), sunscreens limit skin’s ability to synthesise vitamin D3 by absorbing solar radiation. Octocrylene has advantageous physical properties for sunscreen [3]. Because of this, it has become a very common ingredient in sunscreen products, and the amount of octocrylene in various sunscreen products has been given in Table 1. In addition, octocrylene is considered to be safe, but allergic dermatitis has been reported in children [4]. However, it has been reported that sunscreen use may disrupt vitamin D synthesis, and it was discovered that the mean 25(OH)D level in the sunscreen user group was half in comparison with the non-sunscreen user group, creating a conflict over whether or not, in real life situations, daily application of sunscreen may disrupt normal vitamin D synthesis due to the presence of octocrylene [3]. For example, some countries, such as Palau, have banned the use of octocrylene in sunscreen for safety reasons [5].

The United States Food and Drug Administration has approved octocrylene for the use of sun protection factor (SPF). In addition, octocrylene is also used in other products such as shampoos, hair sprays, tannin oils, and conditioners, and octocrylene has become an environmental contaminant found in coastlines, rivers, and lakes all over the world [6,7,8]. However, the concertation of octocrylene in the rivers and waste water has been reported in the range of 5.2–38.0 mg/L, and in zebrafish, androgenic and estrogenic effects of octocrylene have been reported [6]. However, toxicokinetic studies concluded that after systemic absorption of octocrylene, its concentrations in plasma were found to be in the range of 25–100 nM [7,8]. Moreover, studies in zebrafish have already concluded that octocrylene may disrupt normal endocrine hemostasis [9,10]. As per a recent study, octocrylene has been found to disrupt estradiol and prevent the synthesis of spermatozoa in Japanese medaka (Oryziaslatipes) [11]. It has also been found that octocrylene may bioaccumulate, and higher concentrations have been reported in organisms of higher trophic levels [6,10,12,13]. However, it has been observed that octocrylene has the ability to penetrate epidermis and may accumulate in a significant amount due to continued exposure [13,14,15]. However, the toxic profile of octocrylene is still not clear in comparison with other ingredients of sunscreen.

In addition, it has been reported that the use of sunscreen may disrupt vitamin D synthesis [16]. However, more research is needed to clarify any potential harmful consequences of octocrylene on human health. Vitamin D plays a significant role in the homeostasis of the human body. There is not enough information available about the impact of octocrylene on the vitamin D synthesis pathway. To assess the efficacy of octocrylene on the enzyme responsible for the progression of vitamin D synthesis and the vitamin D receptor, we have conducted dynamic and molecular interaction analyses in order to find octocrylene’s potency to bind stably with the vitamin D receptor and other enzymes responsible for the progression of vitamin D synthesis.

## 2. Results

### 2.1. Molecular Docking Analyses of Octocrylene in Comparison with Calcitriol for the Vitamin D Binding Protein

**Figure 2:**
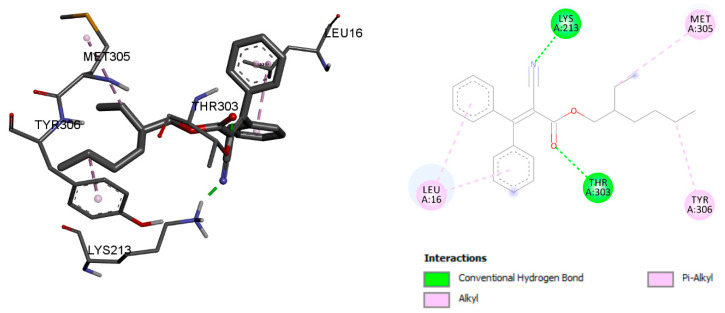
Octocrylene molecular interaction with the vitamin D binding protein.

**Figure 3:**
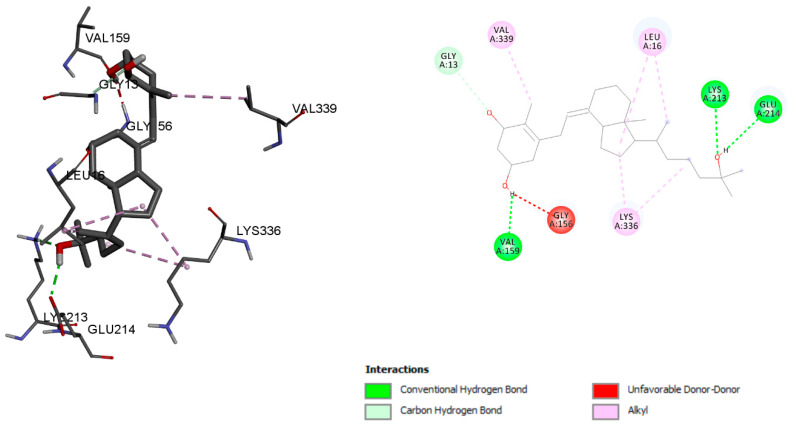
Calcitriol molecular interaction with the vitamin D binding protein.

The binding energy score of octocrylene with the vitamin D binding protein was −8.78 kcal/mol, and the interaction was stabilised with conventional hydrogen bond, Alkyl, and Pi-Alkyl bond interactions with residues LYS 213, THR 303, MET 305, LEU 16, and TYR 3036, respectively (Figure 2). However, calcitriol showed a −9.45 kcal/mol binding energy. LYS 213, GLU 214, and VAL 159 residues were associated with conventional hydrogen bonds, and residues LEU 16, LYS 336, and VAL 339 were associated with Alkyl bonds (Figure 3).

**Figure 4:**
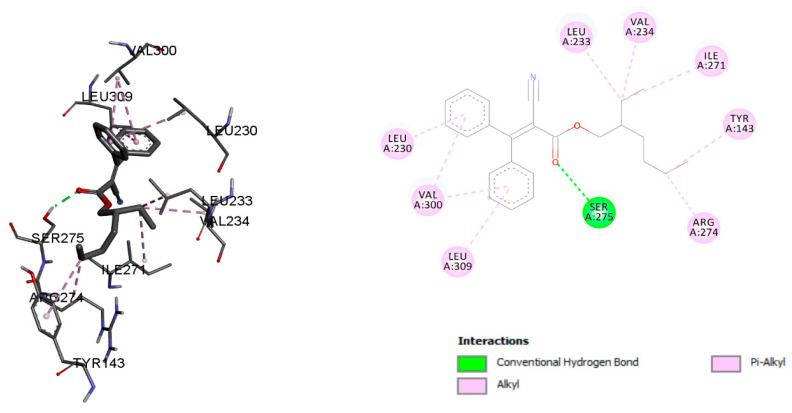
Octocrylene molecular interaction with the vitamin D receptor.

**Figure 5:**
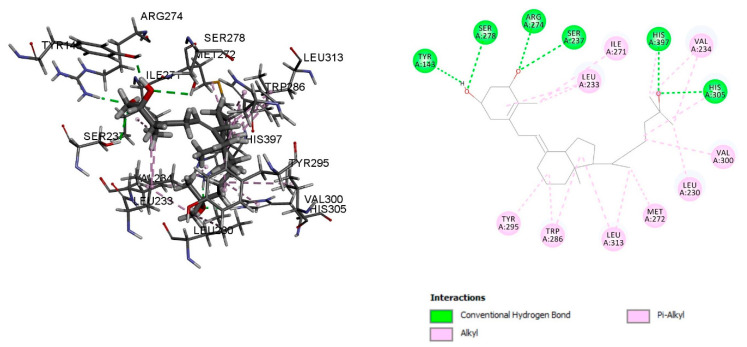
Calcitriol molecular interaction with the vitamin D receptor.

The vitamin D-receptor-related disruption may be responsible for rickets and osteoporosis (Abouzid). Octocrylene showed a binding energy of −8.2 kcal/mol and was stabilized with conventional hydrogen bond residues SER 275 and Alkyl and Pi-Alkyl bonds LEU 230, VAL 300, LEU 309, ARG 274, TYR 143, ILE 271, VAL 234, and LEU 233 (Figure 4). The calcitriol binding energy with the vitamin D receptor was −12.29 kcal/mol, and residues TYR 143, SER 278, ARG 274, SER 237, HIS 397, and HIS 305 were stabilised with conventional hydrogen bonds, Alkyl, and Pi-Alkyl bonds with residues LEU 233, ILE 271, VAL 234, VAL 300, LEU 230, MET 272, LEU 313, TRP 286, and TYR 295 (Figure 5).

**Figure 6:**
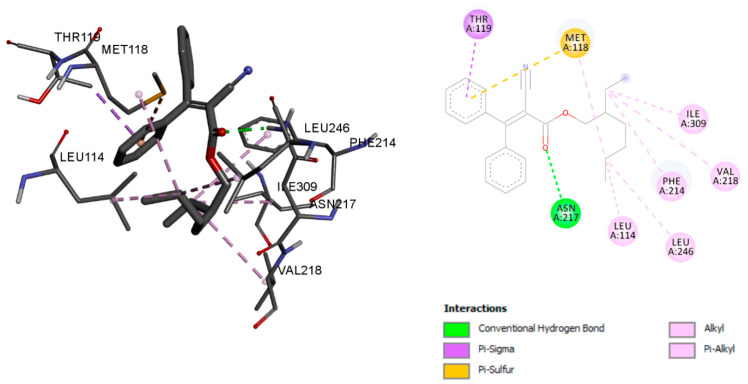
Octocrylene molecular interaction with the enzyme CYP2R1.

**Figure 7:**
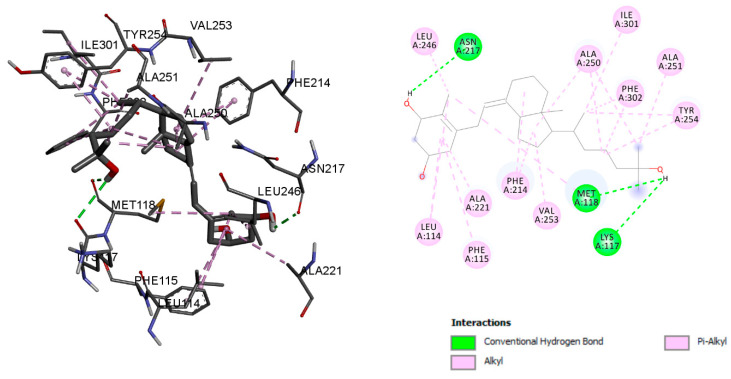
Calcitriol molecular interaction with the enzyme CYP2R1.

The main vitamin D 25-hydroxylase enzyme is called CYP2R1, also known as cytochrome P450 2R1 [17]. Octocrylene has shown a binding energy of −8.96 kcal/mol with CYP2R1, and the calcitriol binding energy was −10.36 kcal/mol. Conventional hydrogen bonds, Alkyl bonds, Pi-Alkyl bonds, Pi-sigma bonds, and Pi-sulphur bonds were used to stabilise octocrylene with residues ASN 217, LEU 114, PHE 214, VAL 218, ILE 309, LEU 246, THR 119, and MET 118, respectively (Figure 6). However, calcitriol formed conventional hydrogen bonds with ASN 217, MET 118, and LYS 117, and residues LEU 246, ALA 250, ILE 301, ALA 251, TYR 254, PHE 302, LEU 114, PHE 115, ALA 221, PHE 214, and VAL 253 were associated with Alkyl and Pi-Alkyl bonds (Figure 7). The details of molecular interaction analyses are given in Table 2.

### 2.2. MD Simulation Analyses

**Figure 8:**
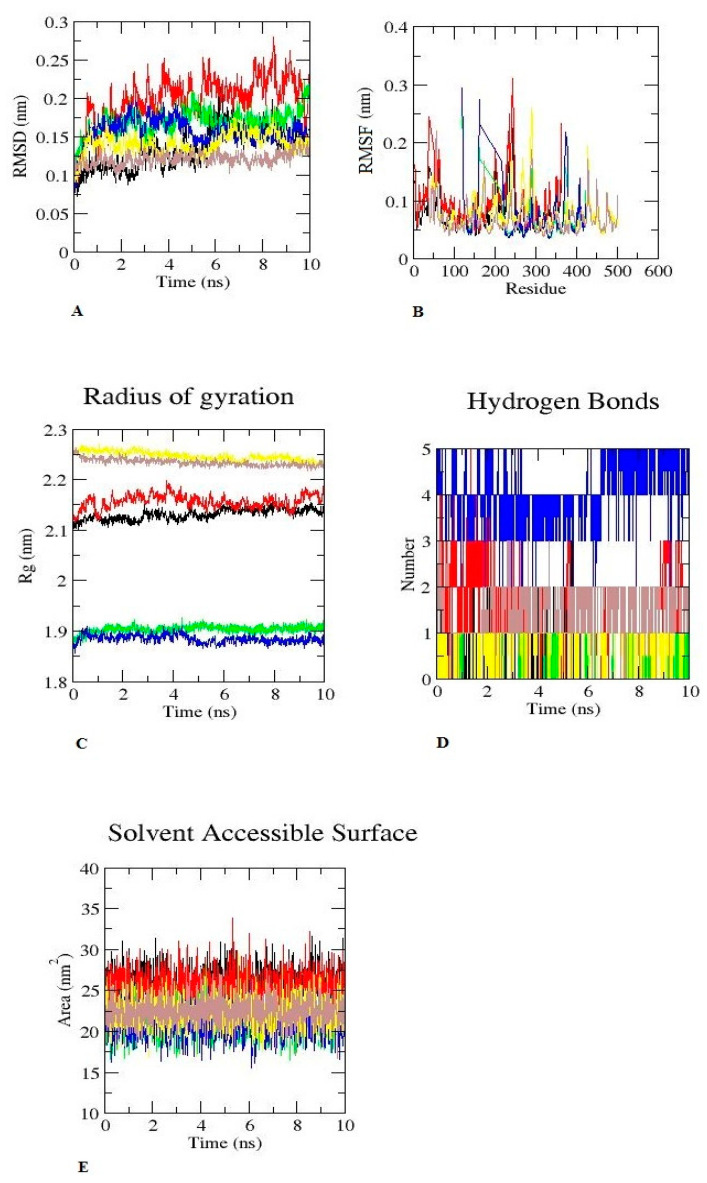
Molecular dynamic (MD) simulation. Black colour is for the vitamin D binding protein+ octocrylene. Red colour is for the vitamin D binding protein + calcitriol. Green colour is for the vitamin D receptor + octocrylene. Blue colour is for the vitamin D receptor + calcitriol. Yellow colour is for the enzyme CYP2R1 + octocrylene. Brown colour is for the enzyme CYP2R1 + calcitriol. (**A**) RMSD for 10 ns. (**B**) RMSF for to 10 ns. (**C**) Radius of gyration corresponds to 10 ns. (**D**) Number of hydrogen bonds between the ligand and the receptor. (**E**) SASA for 10 ns.

For the assessment of octocrylene conformational stability and fluctuation pattern with the vitamin D receptor and the associated enzyme, we have assessed several parameters such as the root-mean-square deviation (RMSD), the root-mean-square fluctuation (RMSF), the radius of gyration (Rg), intermolecular hydrogen bonds, and the solvent-accessible surface area (SASA) in comparison with calcitriol because ligand and protein interaction stability is important in understanding their long-term effects on biological systems. For MD simulations of ligand–protein complexes for 10 ns, we used the GROMACS software. The results of the MD simulation are depicted in Figure 8. It is apparent from the figure that the RMSD value of octocrylene with the vitamin D binding protein was almost in the range of ≤0.05 nm, which means that octocrylene has the ability to undergo stable molecular interactions. Although calcitriol has an RMSD value within the range of 0.05 nm–0.1 nm, this comparison shows that octocrylene’s stability for the vitamin D binding protein is better. Furthermore, the RMSD of octocrylene and calcitriol with the vitamin D receptor was 0.05 nm–0.1 nm. The CYP2R1–octocrylene and CYP2R1–calcitriol RMSD values are in the range of ~0.5 nm. The details are given in Figure 8A.

RMSF can be used to evaluate the flexibility of octocrylene among the residues. The fluctuation pattern of octocrylene with the vitamin D binding protein is similar to that of calcitriol. In addition, the fluctuation of octocrylene with the vitamin D receptor and CYP2R1 is similar to that of calcitriol. Overall, octocrylene showed restricted movement with the respective receptor similar to the native ligand calcitriol (Figure 8B). In addition, for the evaluation of conformational changes during the simulation, the gyration plot of octocrylene and the respective receptor in comparison with the native ligand calcitriol was used. The Rg value of octocrylene with the vitamin D binding protein fluctuated at 2.1 nm and decreased to a minimal value of 2.15 nm, which is similar to calcitriol; the Rg value of octocrylene with the vitamin D receptor fluctuated at 1.9 nm and decreased at ~1.9 nm. The fluctuation of octocrylene was almost similar to, or superimposed with, the native ligand calcitriol. The Rg values of CYP2R1–octocrylene and CYP2R1–calcitriol were superimposed with each other; the Rg value fluctuated at 2.25 nm and decreased at ~2.25 nm only. This smaller radius of gyration demonstrates that octocrylene has similar compactness to calcitriol when bound with the vitamin binding protein, the vitamin D receptor, and the CYP2R1 enzyme responsible for the vitamin D synthesis progression (Figure 8C).

Hydrogen bond interaction analysis was performed to maintain the overall stability of octocrylene with the vitamin binding protein, the vitamin D receptor, and the CYP2R1 enzyme in comparison to calcitriol. This hydrogen bond investigation shows that octocrylene has the ability to form stable interactions with the vitamin binding protein, the vitamin D receptor, and the CYP2R1 enzyme, similarly with calcitriol (Figure 8D). In addition, for the determination of the receptor and ligand complexes with solvents, the solvent-accessible surface area (SASA) was calculated. The SASA indicates the interaction of receptor–ligand complexes with solvents and also represents conformational changes during the MD simulation. The SASA reveals that octocrylene has the ability to form aqueous solutions, similarly with calcitriol, driven by the CHARMM force field 36, 2021 from GROMACS-2019 software. The SASA for octocrylene and calcitriol with the vitamin D binding protein, the octocrylene–vitamin D receptor, and the octocrylene–CYP2R1 were 27 ± 0.5 nm, 20 ± 0.5 nm, and 25 ± 0.5 nm (Figure 8E).

### 2.3. Molecular Mechanics Poisson–Boltzmann Surface Area (MM-PBSA) and Interaction Energy of Octocrylene and Calcitriol

The MM-PBSA gives details about the quantitative estimation of the interaction mechanism of the receptor and ligand molecules. We analysed the binding free energy, van der Waal’s, electrostatic energy, and polar solvation energy. In addition, interaction energy was also calculated to assess total stability analysis. The details are given in Table 3.

### 2.4. ADME (Absorption, Distribution, Metabolism, and Excretion) Analyses

Using the Swiss ADME server, the ADME properties of octocrylene were predicted. Octocrylene demonstrated notable values for numerous characteristics. It might readily replicate the pharmacokinetic characteristics of the calcitriol natural ligand and disrupt vitamin D synthesis. Table 4 provides information about ADME.

## 3. Discussion

Vitamin D deficiency is one of the emerging global health problems. As per the report, almost 1 billion people around the globe are suffering from vitamin D deficiency [18]. There may be several reasons for vitamin D deficiency, such as dietary problems or disease. However, vitamin D deficiency may also be possible in individuals who use sunscreen consistently [18]. In the UVB region, there is a significant overlap between the action spectra for erythema and vitamin D synthesis. Therefore, sunscreen that prevents erythema may also create a nuisance for vitamin D synthesis. However, sunscreen is formulated with various ingredients, among them octocrylene, used as a photosensitizer. In addition, the presence of octocrylene in urine has been reported, which reflects that octocrylene has the ability to deposit in body fluids or tissue [19]. The presence of octocrylene has also been found in marine and freshwater environments because it is widely used in personal care products such as creams, lip care products, makeup, and hair sprays. Octocrylene concentrations in wastewater have been reported to be in the 5.2–38.0 g/L range [6,19,20]. As per toxicokinetic data, the plasma concentration of octocrylene has been reported in the range of 25–100 nM [7,8]. However, the negative impact of octocrylene on human health needs to be further elucidated [20].

We have performed an in silico assessment to understand the interactive potency of octocrylene with the enzyme responsible for the progression of vitamin D synthesis and the vitamin D receptor. With a high binding score, octocrylene interacted well with vitamin D binding protein (1KXP), the vitamin D receptor (1DB1), and the enzyme CYP2R1 (3CZH). The molecular interaction of octocrylene was found to be similar in comparison with calcitriol. The common interactive residues with 1KXP–octocrylene and 1KXP–calciterol; 1DB1–octocrylene and 1DB1–calciterol; and 3CZH–octocrylene and 3CZH–calciterol were LYS 213 with hydrogen bonds, LEU 16 as Alkyl bonds, LEU 233, VAL 234, ILE 271 as Alkyls, Pi-Alkyl bonds, ASN 217 as hydrogen bonds, LEU 114, LEU 246, PHE 214 as Alkyls, and Pi-Alkyl bonds; all interaction patterns reflect that octocrylene has the ability to mimic interaction patterns similar to calcitriol, which is the native ligand for vitamin D synthesis and the vitamin D receptor.

To understand the stability of the octocrylene complex with the respective receptor, we have conducted an MD simulation in comparison with calcitriol. RMSD is commonly used to assess complex stability; if the ligand (octocrylene) attaches to the receptor stably, it may disrupt the normal biochemical pathway. The protein backbone RMSD of octocrylene with the receptor was found to be similar to calcitriol. All these findings demonstrate that octocrylene may bind in a similar manner as calcitriol with the vitamin D binding protein, the enzyme CYP2R1, and the vitamin D receptor and may interfere with the normal synthesis of vitamin D. In addition, conformational changes such as RMSF, gyration, and hydrogen bond patterns were similar to calcitriol. The SASA value of octocrylene was almost similar to calcitriol. The MD approach has been used for the prediction of the toxicity potential of xenobiotics [20]. From the MD trajectories by using MM-PBSA, and we estimated the van der Waal’s energy, electrostatic energy, and solvation energy. These findings may be useful in understanding how octocrylene behaves molecularly in comparison to calcitriol [21].

Regarding toxicological considerations, octocrylene has been considered as an endocrine disrupter and has the ability to create metabolic nuisances in fish models. In effect in January 2020, octocrylene has been disallowed in sunscreen and cosmetic goods in a number of countries, including the Republics of Palau and the Marshall Islands, as well as the U.S. Virgin Islands. The ADMET parameters of octocrylene categorised it as having a high potential for gastrointestinal absorption, and it can also cross the blood–brain barrier. Considering all the findings, it can be concluded that octocrylene has the ability to cause stable molecular interactions, resulting in abnormalities in the synthesis of vitamin D and toxicities.

## 4. Materials and Methods

### 4.1. Structural Preparation for Molecular Docking

The Protein Data Bank (PDB) was used to extract the X-ray crystal structure of the enzyme responsible for the progression of vitamin D synthesis and the vitamin D receptor. The details are given in Table 5.

With regard to the vitamin D receptor, octocrylene was relocked utilising the Auto Dock 4.2 tool [22,23]. For optimal molecular docking, water molecules were removed from the crystallographic structure. In addition, solvation parameters, Kollman combined charges, and hydrogen were added to the protein in ideal geometry. The molecular docking parameter, van der Waal’s force, was assigned to the protein in ideal geometry; the protein structure was saved in the PDBQT format, and torsion was fixed for the complex. The active site for the ligand was recognised by PDBsum, and the grid was fixed around the recognised active site for precise molecular docking. The docking parameters files (DPF) and grid parameters files (GPF) of the ligand AutoDock tools were used. Furthermore, to assess protein–ligand binding conformations, the Lamarckian Genetic Algorithm was used [22]. The 50 confirmation poses were used to generate the binding energy of the ligand and protein interaction. Among the 50 conformational poses, the protein–ligand complex with the highest binding energy was selected for molecular dynamic (MD) studies so that the stability of the molecular interactions of octocrylene with the respective protein could be evaluated. For the visualisation of protein and ligand complexes, Discovery Studio 16 molecular visualisation software was used [24].

### 4.2. Molecular Dynamic (MD) Simulation for Stability Assessment of Octocrylene with Respective Protein

Using the GROMACS-2019 programme, an MD simulation was run for a duration of 10 ns to assess the structural and dynamic changes in the respective receptor and enzyme of the vitamin D synthesis pathway. The Charmm 36 force field 2021 was used to parameterize all atoms [25]. For the solvation of protein–ligand complexes, simple point-charged water molecules and counter ions (Cl or Na) were applied for neutralization [26]. Energy minimization was used to remove van der Waal’s contacts between the atoms. The complex was equilibrated for 10 ns in two phases. The first phase was a constant number of particles, volume, and temperature (NVT), which was assembled with endothermic and exothermic processes and is exchangeable with a thermostat. The constant number of particles, pressure, and temperature (NPT) was the second phase. Both phases were assembled at 310 K at the human body temperature along with constant pressure followed by the application of the Linear Constraint Solver (LINCS) algorithm to constrain covalent bonds. The MD was conducted for a duration of 10 ns to analyse the stability of octocrylene with the respective receptor and enzyme of vitamin D.

### 4.3. Analysis of Trajectory

Using the GROMACS g_rmsd plugin, the simulation results were represented in terms of root-mean-square deviation (RMSD). The system (protein–ligand complex) was also determined by the radius of gyration (RG), root-mean-square fluctuation (RMSF), and a number of intermolecular hydrogen bonds using the g_gyrate, g_rmsf, and g_hbond plugins of GROMACS, respectively [27].

### 4.4. Interaction Energy of Octocrylene and Calcitriol

Using GROMACS coulombic interaction energy and the short-range Lennard–Jones energy, the nonbonded interaction energy between ligand and substrate was calculated in order to evaluate the intensity of the interaction between the ligand and the specific receptor [28].

### 4.5. Molecular Mechanics Poisson–Boltzmann Surface Area (MM-PBSA)

To determine free binding energy, the MM-PBSA protocol was used. The van der Waal’s, solvation energy, and electrostatic energy were also calculated for the octocrylene in comparison with calcitriol as per the given methods:

ΔG bind = G complex − (G Protein + G Ligand)

ΔG is the binding free energy of the octocrylene + respective receptor and the enzyme of the vitamin D synthesis pathway.

G ligand represents octocrylene and calcitriol.

G protein represents the binding energy of the respective receptor and the enzyme of the vitamin D synthesis pathway [29,30].

### 4.6. ADME Analyses

The comparative pharmacokinetic characteristics of octocrylene and calcitriol were evaluated using the Swiss ADME online tool (http://www.swissadme.ch, accessed on 14 August 2022, run by the Swiss Institute of Bioinformatics [SIB], Lausanne, Switzerland) [24].

## 5. Conclusions

Octocrylene interacts stably with the vitamin D binding protein, the vitamin D receptor, and the enzyme CYP2R1, indicating a high risk of vitamin D abnormalities. In addition, octocrylene may cross the blood–brain barrier and has a potent skin absorption ability. Octocrylene, by capturing the binding site of a natural ligand such as calcitriol, may act as a barrier to the proper function of the required ligand calcitriol, resulting in abnormal vitamin D synthesis in the human body. These dynamics, molecular interactions, and the MMPBSA-based in silico assessment may be considered as a reference for clinical and experimental studies.

## Figures and Tables

**Table 1:** Example of brand and concentration of octocrylene.

SN	Product Name	Octocrylene Concentration	Information Source
1	Nivea Sun Item weight 200 mL	Octocrylene 50 mg	Available online: https://www.amazon.de/Nivea-Feuchtigkeits-Sonnenlotion-200mL-Badartikel/dp/B000PE8B16 (Accessed on 14 August 2022)
2	Bana Boat Sport performance Item weight 172 gm	Octocrylene 13.76 mg	Available online: https://www.amazon.in/Banana-Boat-Performance-Sunscreen-Lotion/dp/B00UNCZ1SG (Accessed on 14 August 2022)
3	Bana Boat Item weight 18 gm	Octocrylene 1.33 mg	Available online: https://www.amazon.in/Banana-Boat-Sunscreen-Perfomance-Spectrum/dp (Accessed on 14 August 2022)
4	Neutrogena Sunscreen SPF 70—6.7 Ounces Lotion 190 gm	Octocrylene 1.9 mg	Available online: https://www.amazon.in/Neutrogena-Defense-Sunscreen-Lotion-Spectrum/dp/B01N1IJA4N (Accessed on 14 August 2022)
5	Coppertone Sport Clear SPF 30	Octocrylene 5.98 mg	Available online: https://www.amazon.in/Coppertone-Sport-Continuous-Spray-6- (Accessed on 14 August 2022)
6	Coppertone Sport Clear SPF 50	Octocrylene 5.98 mg	Avialble online: https://www.amazon.com/Coppertone-Sunscreen-Lotion (Accessed on 14 August 2022)

**Table 2:** Molecular interaction analyses.

Ligands	Amino Acid Residues Involved in Hydrogen Bonds	Docking Final Intermolecular Energy (ΔG) = vdW + Hbond + Desolv Energy (kcal/mol)	Inhibition Constant (Ki)	Protein
Octocrylene	LYS 213 THR 303	−11.52	365.25 nM	1KXP
Calcitriol	LYS 213 VAL 159 GLU 214	−11.71	117.72 nM	
Octocrylene	SER 275	−11.15	979.57 nM	1DB1
Calcitriol	SER 278 TYR 143 ARG 274 SER 237 HIS 397 HIS 305	−8.73	2.99 µM	
Octocrylene	ASN 217	−11.9	271.01 nM	3CZH
Calcitriol	ASN 217 MET 118 LYS 117	−13.03	25.63 nM	

**Table 3:** Molecular mechanics Poisson–Boltzmann surface area (MM-PBSA) and interaction energy.

Parameters	Octocrylene + Vitamin D Binding Protein (1KXP)	Calcitriol + Vitamin D Binding Protein (1KXP)	Octocrylene + Vitamin D Receptor (1DB1)	Calcitriol + Vitamin D Receptor (1DB1)	Octocrylene + Enzyme CYP2R1 (3CZH)	Calcitriol + Enzyme CYP2R1 (3CZH)
van der Waal’s energy	−37.87 ± 0.133 kcal/mol	−43.35 ± 0.16 kcal/mol	−49.28± 0.07 kcal/mol	−55.09 ± 0.09 kcal/mol	−49.62 ± 0.08 kcal/mol	−54.75 ± 0.08 kcal/mol
Electrostatic energy	−1.8019 ± 0.03 kcal/mol	−1.699 ± 0.02 kcal/mol	−0.477 ± 0.0094 kcal/mol	−41.82± 0.1 kcal/mol	−0.752 ± 0.0087 kcal/mol	−49.62 ± 0.08 kcal/mol
Solvation energy	−1.8019 ± 1.2 kcal/mol	−1.91 ± 0.01 kcal/mol	−5.05 ± 0.009 kcal/mol	−41.82 ± 0.1 kcal/mol	−5.009 ± 0.0093 kcal/mol	−5.11 ± 0.008 kcal/mol
Binding free energy	−41.82 ± 0.1 kcal/mol	−46.96 ± 0.17 kcal/mol	−54.81 ± 0.07 kcal/mol	−63.25 ± 0.095 kcal/mol	−55.37 ± 0.086 kcal/mol	−61.06 ± 0.08 kcal/mol
Interaction Energy	Coul-SR: −58.22 KJ/mol	Coul-SR: −47.91 KJ/mol	Coul-SR: −21.23 KJ/mol	Coul-SR: −106.515 KJ/mol	Coul-SR: −38.62 KJ/mol	Coul-SR: −33.97 KJ/mol
	LJ-SR: −141.58 KJ/mol	LJ-SR: −160.619 KJ/mol	LJ-SR: −185.137 KJ/mol	LJ-SR: −204.88 KJ/mol	LJ-SR: −186.002 KJ/mol	LJ-SR: −205.138 KJ/mol

**Table 4:** Important ADME and QSAR tool box points of Octocrylene.

SN	ADME Parameters	Results
1	Molecular weight	361.48 g/mol
2	GI absorption	High
3	BBB permeant	Yes
4	Log *K*_p_ (skin permeation)	−3.44 cm/s
5	CYP1A2 inhibitor	Yes
6	CYP2C19 inhibitor	Yes
7	CYP2C9 inhibitor	Yes
8	CYP2D6 inhibitor	Yes
9	CYP3A4 inhibitor	Yes

**Table 5:** Information about the protein and ligands used in the study.

Protein Data Bank	Ligand	Control
Vitamin D binding protein (1KXP)		
Vitamin D receptor (1DB1)	Octocrylene	Calcitriol
Enzyme CYP2R1 (3CZH)	PubChem Id 22571	PubChem Id 5280453

## Data Availability

The authors confirm that the data supporting the study’s findings are included in the article.
